# Honey Bee Dopamine and Octopamine Receptors Linked to Intracellular Calcium Signaling Have a Close Phylogenetic and Pharmacological Relationship

**DOI:** 10.1371/journal.pone.0026809

**Published:** 2011-11-11

**Authors:** Kyle T. Beggs, Joel D. A. Tyndall, Alison R. Mercer

**Affiliations:** 1 Department of Zoology, University of Otago, Dunedin, New Zealand; 2 School of Pharmacy, University of Otago, Dunedin, New Zealand; New Mexico State University, United States of America

## Abstract

**Background:**

Three dopamine receptor genes have been identified that are highly conserved among arthropod species. One of these genes, referred to in honey bees as *Amdop2*, shows a close phylogenetic relationship to the a-adrenergic-like octopamine receptor family. In this study we examined in parallel the functional and pharmacological properties of *Am*DOP2 and the honey bee octopamine receptor, *Am*OA1. For comparison, pharmacological properties of the honey bee dopamine receptors *Am*DOP1 and *Am*DOP3, and the tyramine receptor *Am*TYR1, were also examined.

**Methodology/Principal Findings:**

Using HEK293 cells heterologously expressing honey bee biogenic amine receptors, we found that activation of *Am*DOP2 receptors, like *Am*OA1 receptors, initiates a rapid increase in intracellular calcium levels. We found no evidence of calcium signaling via *Am*DOP1, *Am*DOP3 or *Am*TYR1 receptors. *Am*DOP2- and *Am*OA1-mediated increases in intracellular calcium were inhibited by 10 µM edelfosine indicating a requirement for phospholipase C-β activity in this signaling pathway. Edelfosine treatment had no effect on *Am*DOP2- or *Am*OA1-mediated increases in intracellular cAMP. The synthetic compounds mianserin and epinastine, like *cis*-(Z)-flupentixol and spiperone, were found to have significant antagonist activity on *Am*DOP2 receptors. All 4 compounds were effective antagonists also on *Am*OA1 receptors. Analysis of putative ligand binding sites offers a possible explanation for why epinastine acts as an antagonist at *Am*DOP2 receptors, but fails to block responses mediated via *Am*DOP1.

**Conclusions/Significance:**

Our results indicate that *Am*DOP2, like *Am*OA1, is coupled not only to cAMP, but also to calcium-signalling and moreover, that the two signalling pathways are independent upstream of phospholipase C-β activity. The striking similarity between the pharmacological properties of these 2 receptors suggests an underlying conservation of structural properties related to receptor function. Taken together, these results strongly support phylogenetic analyses indicating that the *Am*DOP2 and *Am*OA1 receptor genes are immediate paralogs.

## Introduction

**Figure 1:**
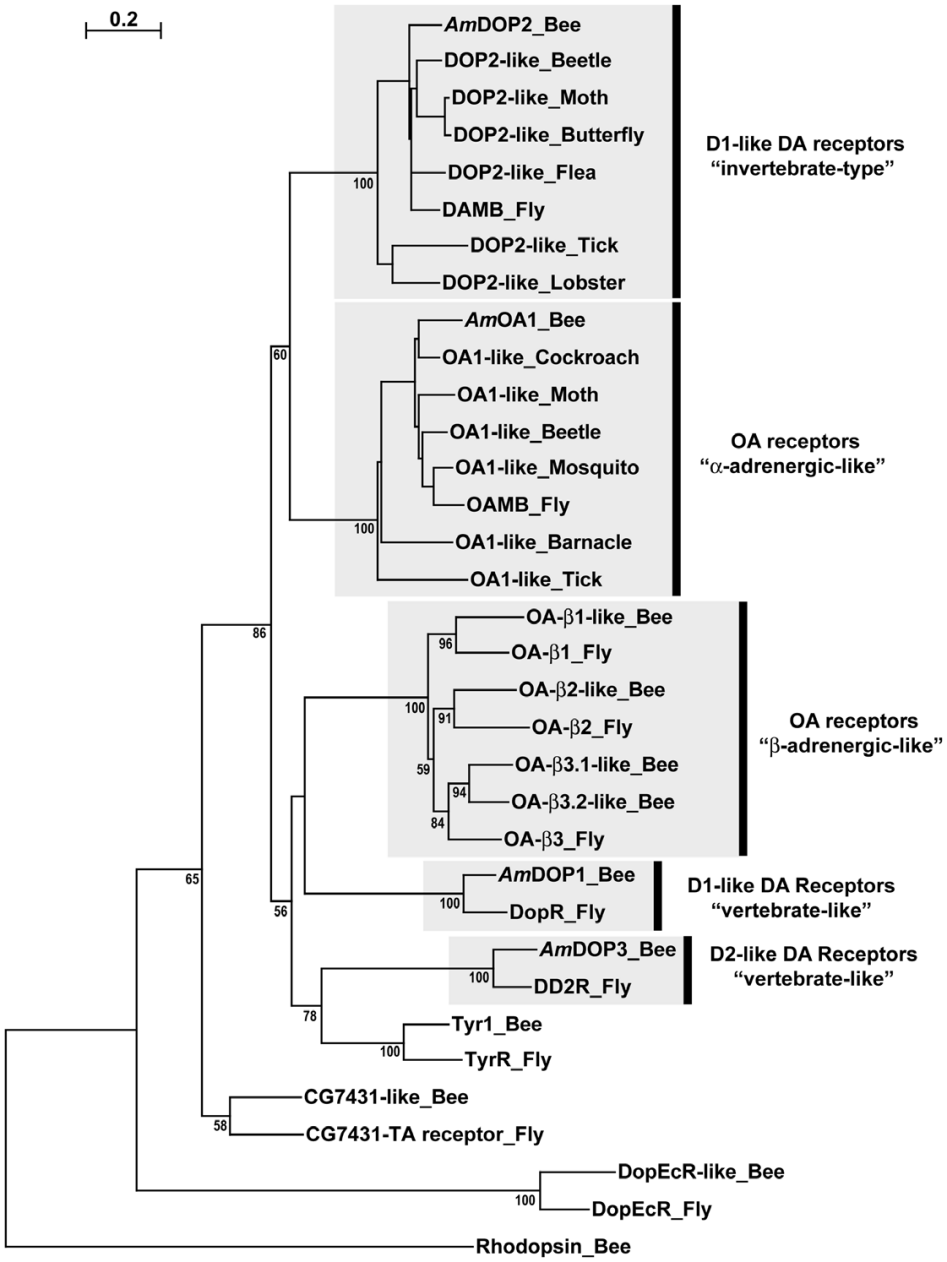
Phylogram of selected arthropod amine receptor sequences. A phylogenetic analysis showing known invertebrate-type DA receptors and α-adrenergic-like OA receptors and their relationship to other DA, OA and TA receptors from *Apis mellifera* and *Drosophila melanogaster*. The honey bee rhodopsin protein is used as an outlier. Conserved regions of receptor protein sequences were aligned using ClustalW2 software (version 2.0.10) using the default settings (http://ww.ebi.ac.uk/Tools/clustalw2/). Phylograms were prepared as described in [30] by a 1000 trial N-J bootstrap analysis, and using ClustalX software (version 2.0). Resulting bootstrap scores are displayed on selected nodes as percentages values, if greater than 50%.

Some invertebrate and vertebrate dopamine (DA) receptor types demonstrate a strong phylogenetic relationship that is reflected in an apparent conservation of common functional properties [1], [2], [3]. For example, D2-like DA receptors in arthropods exhibit significant homology in primary amino acid sequence with vertebrate D2-like dopamine receptors [4], [5], [6] and in arthropods, as in vertebrates, activation of D2-like receptors generally inhibits adenylyl cyclase activity leading to a reduction in intracellular levels of cAMP [1], [3]. However, phylogenetic analyses indicate that at least one DA receptor type may be specific to invertebrate species [1], [7]. The presence of this ‘invertebrate-type’ DA receptor (Figure 1) raises a number of interesting questions about the origin, function and role of this receptor protein in the invertebrate nervous system.

The first of these invertebrate-type DA receptors to be functionally described was the *Drosophila* DAMB/Dop99B receptor [8], [9]. Orthologues of this receptor have subsequently been described in several arthropod species [10], [11], [12], [13], including in honey bees (*Am*DOP2, [7]). All of the invertebrate-type DA receptors examined so far have been found to be positively coupled to adenylyl cyclase and hence are often described as D1-like DA receptors [1]. Interestingly, stimulation of cells expressing the DAMB/Dop99B receptor with DA also induces a rapid, transient increase in intracellular calcium (Ca^2+^) levels [9], [14], [15]. However, this property has yet to be demonstrated in other invertebrate-type DA receptors.

One intriguing feature of invertebrate-type DA receptors is that their primary amino acid sequences, when compared to other invertebrate biogenic amine receptors, show highest homology to an octopamine (OA) receptor type [1], [7], [16], [17], [18] described as being ‘α-adrenergic-like’ [19]. The first α-adrenergic-like OA receptor was described in a pond snail [20] and orthologues that have been identified subsequently include the *Drosophila* OAMB receptor [14], honey bee *Am*OA1 receptor [21], cockroach Pa oa_1_ receptor [22] and silkworm *Bm*OAR1 [23] receptor. Functional studies of cells expressing α-adrenergic-like OA receptor orthologues have consistently found that activation by OA results in a rapid, transient rise in intracellular Ca^2+^ concentrations, but also an increase in intracellular levels of cAMP [14], [16], [20], [21], [22], [23]. Interestingly, depletion of intracellular Ca^2+^ with BAPTA-AM was found to have no significant effect on cAMP signaling mediated either, via DAMB/Dop99B receptors [15] or Pa oa_1_-receptors [22] indicating that both of these receptor types mediate their effects on cAMP via a pathway independent of the Ca^2+^ signaling pathway. However this concept of pathway independence has also been challenged [16], [19], [21], with increases in cAMP levels suggested to be the result of Ca^2+^-induced adenylyl cyclase activity.

Phylogenetic models have indicated that the invertebrate-type DA receptors and the α-adrenergic-like OA receptors are immediate paralogs [17], [18]. However, as such models may contain inherent weaknesses in their ability to discern distant evolutionary relationships correctly [24], evidence at a functional level is required also to confirm the existence of such relationships. In this study we examined the functional and pharmacological properties of the invertebrate-type DA receptor from honey bee, *Am*DOP2, and the honey bee α-adrenergic-like octopamine receptor, *Am*OA1, as representative examples of each of the two receptor types (Figure 1). Analysis of these two receptors in parallel enabled us to test predictions arising from phylogenetic modelling.

Our study provides evidence that agonist activation of either, *Am*DOP2 or *Am*OA1 receptors results in stimulation of cAMP and Ca^2+^ signaling pathways, and that these pathways are activated independently upstream of PLCβ activity. We also show that there are marked similarities in the pharmacological properties of these two receptor types, a finding supported further by comparisons with other related receptor types. The homology we find in the functional and pharmacological properties of *Am*DOP2 and *Am*OA1 receptors supports the phylogenetic model of these receptor genes as being immediate paralogs.

## Results

### *Am*DOP2 receptors couple to Ca^2+^ signaling pathways

**Figure 2:**
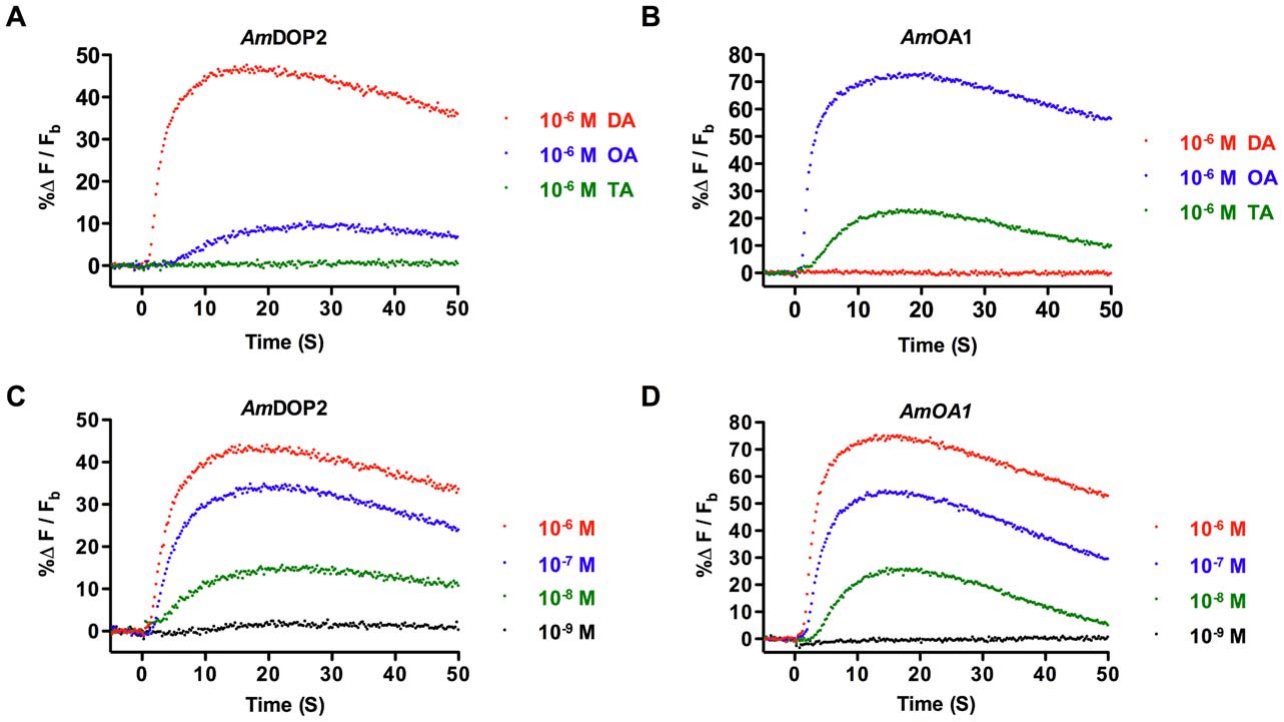
Agonist-induced changes in intracellular Ca^2+^ levels in HEK293 cells expressing either *Am*DOP2 receptors or *Am*OA1 receptors. Cells were preloaded with the [Ca^2+^]_I_ reporter dye Fluo-4 and monitored for agonist-induced changes in fluorescence signal. The data shown are from a single trial and are representative of results obtained from 3 independent trials at each agonist concentration (with two internal replicates per trial), and over 30 trials using 1 µM. Changes in fluorescence examined in the following: (A) *Am*DOP2-expressing HEK293 cells exposed to 1 µM DA, OA or TA; (B) *Am*OA1-expressing HEK293 cells exposed to 1 µM DA, OA or TA; (C) *Am*DOP2-expressing HEK293 cells exposed to DA at the concentrations indicated to the right of Figure 1C; (D) *Am*OA1-expressing HEK293 cells exposed to OA at the concentrations indicated to the right of Figure 1D.

We found that exposure of *Am*DOP2-expressing HEK293 cells to 1 µM DA initiated a rapid increase in intracellular Ca^2+^ levels, with maximum response amplitude occurring 15 to 20 seconds post injection (Figure 2A). Exposure of *Am*DOP2-expressing cells to 1 µM OA also initiated a Ca^2+^ response, but responses to this amine were smaller in amplitude and slower to reach peak levels (approximately 25 s) than responses to DA (Figure 2A). Exposure to 1 µM tyramine (TA) had no observable effect on *Am*DOP2-expressing cells (Figure 2A). Control cells expressing the β-Gal reporter protein showed no Ca^2+^ response to DA, OA or TA (data not shown). The Ca^2+^ response of *Am*DOP2-expressing HEK293 cells to DA was dose-dependent (Figure 2C).

For comparison we also examined *Am*OA1-expressing HEK293 cells. *Am*OA1-mediated Ca^2+^ signaling has been identified previously in HEK293 cells using single cell monitoring [21] and we found also that *Am*OA1-expressing HEK293 cells showed a rapid increase in intracellular Ca^2+^ levels in response to 1 µM OA (Figure 2B). Responses to 1 µM OA peaked approximately 15 to 20 seconds post injection and then declined steadily. *Am*OA1-expressing HEK293 cells also showed a smaller amplitude response to 1 µM TA, but no response to 1 µM DA (Figure 2B). The Ca^2+^ response of *Am*OA-expressing cells to OA was dose-dependent (Figure 2D).

### *Am*DOP2 receptors and *Am*OA1 receptors couple to cAMP signaling via a PLCβ-independent pathway

**Figure 3:**
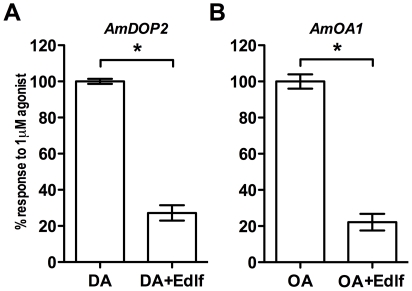
PLCβ activity is required for *Am*DOP2- and *Am*OA1-mediated intracellular Ca^2+^ signaling. Treatment with edelfosine (edlf) was found to inhibit *Am*DOP2 (*P*<0.0001) and *Am*OA1 (*P* = 0.0002) mediated Ca^2+^ signaling. HEK293 cells expressing either *Am*DOP2 receptors (A) or *Am*OA1 receptors (B) were loaded with the intracellular Ca^2+^ reporter dye Fluo-4, with or without the inclusion of 10 µM edelfosine (Edlf) in the loading buffer. Cells were subsequently exposed to a 1 µM concentration of agonist and the maximal ΔF/F_b_ in following 50 s period determined. Data are normalized to the percentage response observed in cells not treated with edelfosine, and are the result of three independent experiments, with two internal replicates per experiment. Error bars represent the SEM. Statistical significance was determined using Student's two-tailed *t* tests.

To determine if *Am*DOP2 and *Am*OA1 receptors are coupled to intracellular Ca^2+^ signaling via a PLCβ-mediated pathway we investigated the effect of the PLC inhibitor, edelfosine [25]. Using HEK293 cells expressing either *Am*DOP2 receptors (Figure 3A) or *Am*OA1 receptors (Figure 3B), we found that treatment with 10 µM edelfosine significantly reduced the amplitude of Ca^2+^ signals elicited by 1 µM DA and 1 µM OA, respectively. These results indicate that PLCβ activity is part of the signaling pathway that leads to increases in intracellular Ca^2+^ levels resulting from *Am*DOP2- or *Am*OA1-receptor activation.

**Figure 4:**
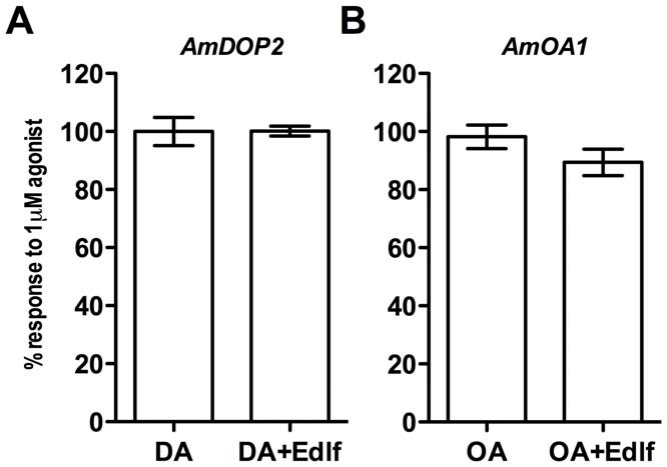
PLCβ activity is not required for *Am*DOP2- or *Am*OA1-mediated intracellular cAMP signaling. Treatment with edelfosine (edlf) had no significant effect on [cAMP]_i_ signaling mediated via *Am*DOP2 receptors (*P* = 0.9751), or via *Am*OA1 receptors (*P* = 0.2224). HEK293 cells were co-transfected with expression constructs for either the *Am*DOP2 receptor (A) or *Am*OA1 receptor (B), and a CRE-luciferase reporter construct. Cells were treated with either 1 µM agonist (DA and OA respectively) or 1 µM agonist and 10 µM edelfosine (edlf). Data are normalized to the response observed in cells not treated with edelfosine, and the mean of three independent experiments within which, each treatment was tested twice. Error bars represent the SEM. Statistical significance was determined using Student's two-tailed *t* tests.

To determine whether *Am*DOP2- and/or *Am*OA1-mediated increases in intracellular cAMP levels require PLCβ activity we tested the effects of edelfosine on responses to DA and OA in *Am*DOP2- and *Am*OA1-expressing HEK293 cells, respectively. We found that in contrast to its effects on Ca^2+^ signaling, treatment with 10 µM edelfosine had no significant effect on agonist-induced changes in levels of cAMP mediated by *Am*DOP2 or by *Am*OA1 (Figures 4A,B). These results suggest that *Am*DOP2 receptors and *Am*OA1 receptors couple to cAMP signaling via a PLCβ-independent pathway.

### *Am*DOP2 and *Am*OA1 receptors have similar pharmacological properties

To compare the pharmacological profiles of *Am*DOP2 and *Am*OA1 receptors we tested four amine-receptor antagonists: *cis*-(Z)-flupentixol, spiperone, mianserin and epinastine. *Cis*-(Z)-flupentixol and spiperone have been used extensively in insects as dopamine-receptor antagonists, whereas mianserin and epinastine are most commonly used in insects as octopamine receptor antagonists (see Discussion).

**Figure 5:**
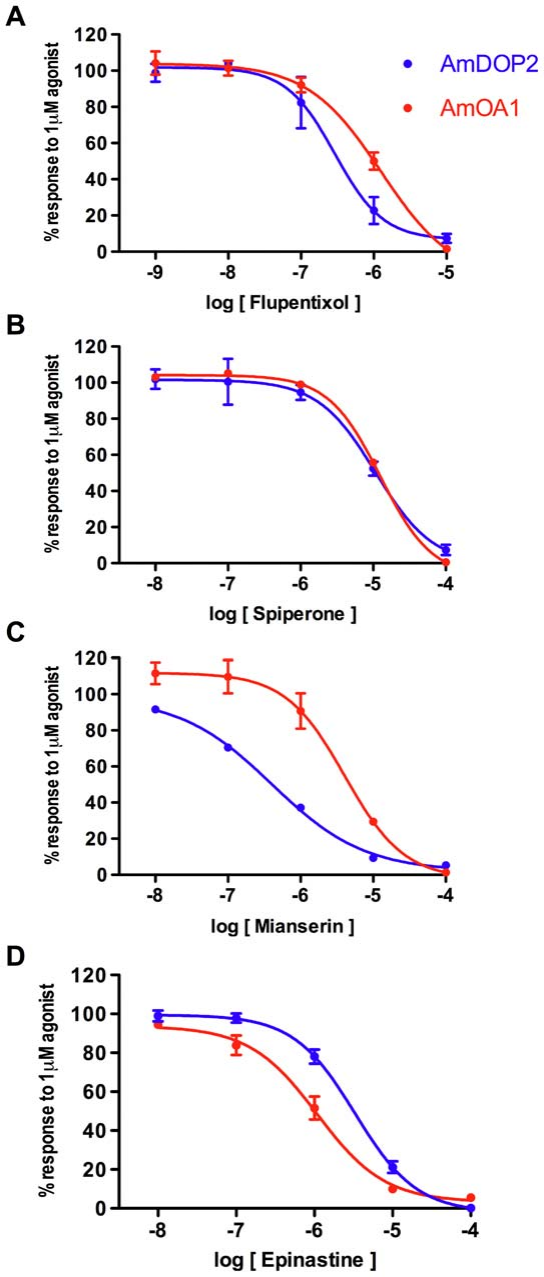
Effects of amine-receptor antagonists on cAMP responses mediated via *Am*DOP2 and *Am*OA1 receptors. HEK293 cells were co-transfected with expression constructs for either the *Am*DOP2 receptor (blue symbols) or *Am*OA1 receptor (red symbols) and a CRE-luciferase reporter construct. Cells were treated with 1 µM agonist (DA or OA respectively) or 1 µM agonist and either *cis*-(Z)-flupentixol (A), spiperone (B), mianserin (C) or epinastine (D), at a range of concentrations indicated in the figure. Due to evidence of significant cell toxicity, *cis*-(Z)-flupentixol was not tested at a concentration higher than 10 µM. Data are normalized to the response observed in cells treated with agonist alone (not shown), and are the result of two independent experiments within which, each treatment was tested twice. Error bars (estimated SEM) are included to provide an indication of consistency between experiments. Dose response curves were determined by non-linear regression using GraphPad Prism software for Macintosh version 5.0b.

**Figure 6:**
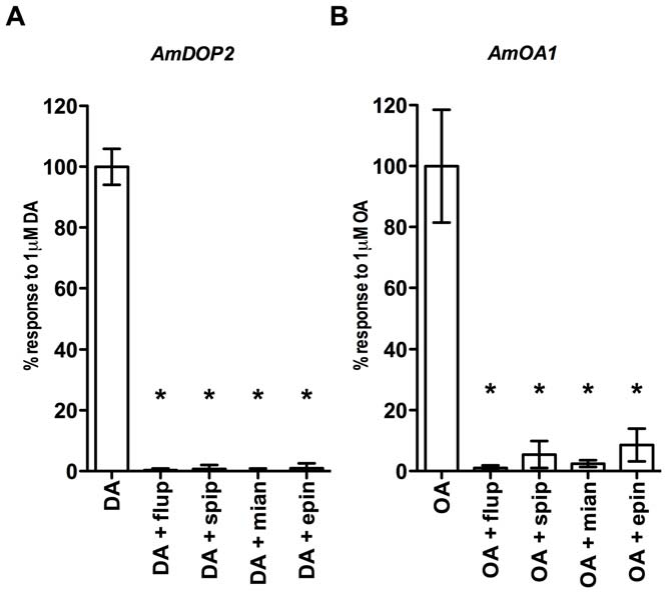
Effect of antagonists on *Am*DOP2- and *Am*OA1-mediated intracellular Ca^2+^ signaling. HEK293 cells expressing either *Am*DOP2 receptors (A) or *Am*OA1 receptors (B) were loaded with the intracellular Ca^2+^ reporter dye Fluo-4 and exposed to 1 µM agonist (DA and OA respectively), or 1 µM agonist and either 10 µM cis-(Z)-flupentixol, spiperone, mianserin or epinastine and the maximal ΔF/F_b_ over following 50 second period determined. Data are normalized to the response observed in cells treated with agonist alone, and are the result of three independent experiments with two internal replicates per experiment. Error bars represent the SEM. Statistical significance was determined using one-way ANOVA and Dunnett's multiple comparison test, with treatment with agonist alone used as the control column. F_4,10_ = 239.1, P<0.0001 for (A); F_4,10_ = 35.95, P<0.0001 for (B).

We began by investigating the effects of each of these compounds on cAMP responses generated through the activation of *Am*DOP2 receptors and *Am*OA1 receptors. Cells expressing *Am*DOP2 or *Am*OA1 receptors were exposed to 1 µM DA or 1 µM OA respectively, together with one of the four putative antagonists (Figure 5). We found that irrespective of whether cAMP responses were mediated via *Am*DOP2 receptors or via *Am*OA1 receptors they could be blocked in a dose-dependent way by all 4 antagonists (Figure 5). Next we tested the effect of a 10 µM concentration of each of these antagonists on *Am*DOP2- and *Am*OA1-mediated Ca^2+^ signals. Again we found that all four compounds acted as effective antagonists of responses mediated by *Am*DOP2 receptors, as well as by *Am*OA1 receptors (Figure 6).

**Figure 7:**
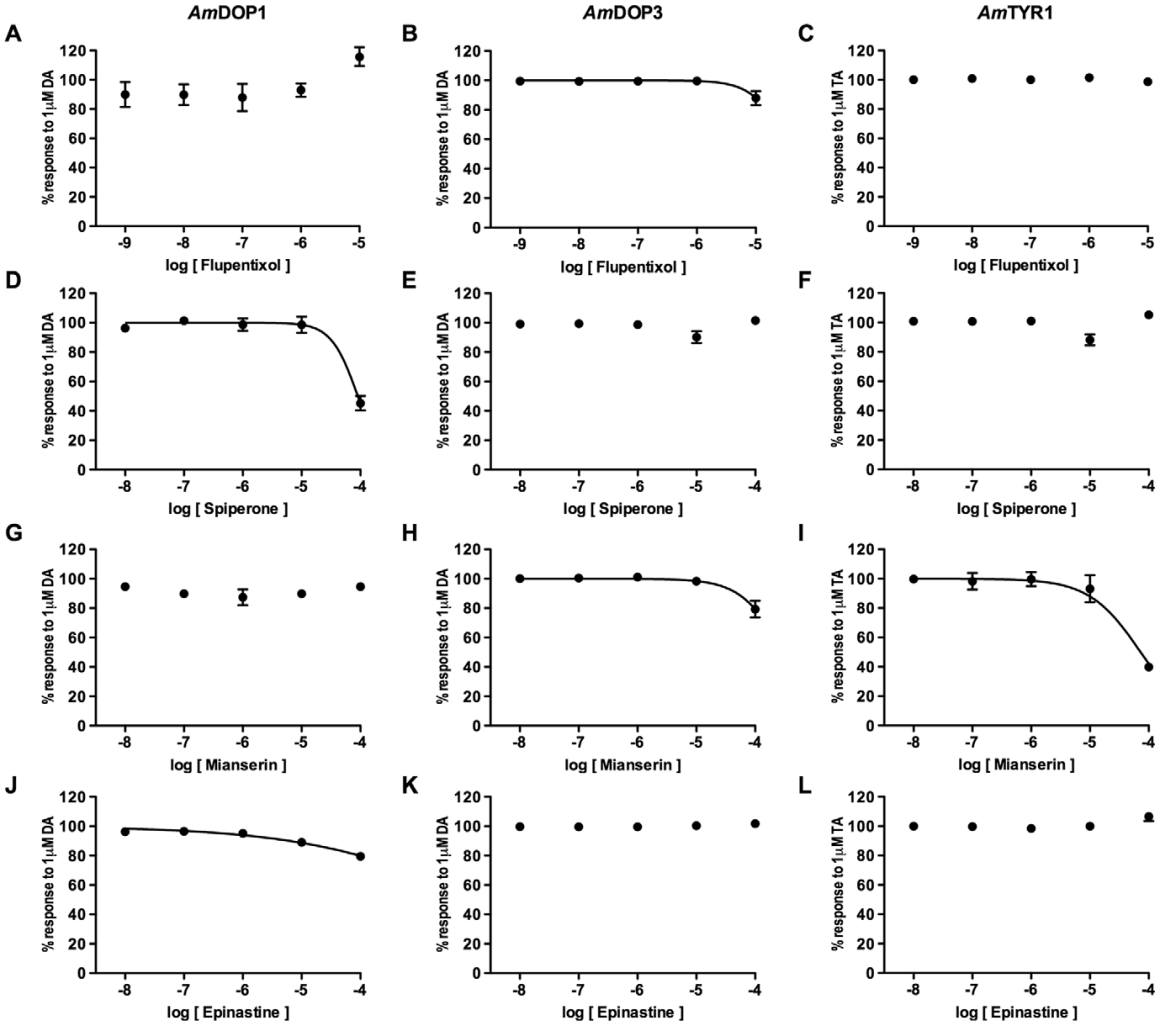
Pharmacological profile of *Am*DOP1, *Am*DOP3 and *Am*TYR1 receptors. HEK293 cells were co-transfected with expression constructs for one of the following amine receptors, the *Am*DOP1 receptor (A, D, G, J), *Am*DOP3 receptor (B, E, H, K), or *Am*TYR1 receptor (C, F, I, L) and a CRE-luciferase reporter construct. Cells were treated with 1 µM agonist, or with 1 µM agonist together with one of the following antagonists: *cis*-(Z)-flupentixol (A, B, C), spiperone (D, E, F), mianserin (G, H, I) or epinastine (J, K, L) at concentrations indicated in each figure. For assaying the activity of *Am*DOP3 and *Am*TYR1 receptors, both of which reduce intracellular cAMP levels, basal cAMP levels in test cells were elevated by inclusion of a nonsaturating concentration of the adenylyl cyclase stimulant, forskolin (100 nM). Data are normalized to the percentage response observed in cells treated with agonist alone (not shown), and are the result of two independent experiments within which, each treatment was tested twice. Error bars represent the estimated SEM. Dose response curves were determined by non-linear regression using GraphPad Prism software for Macintosh version 5.0b, and displayed when the resulting curves were unambiguous.

To assess further the specificity of the 4 antagonists, we examined their actions on changes in intracellular cAMP mediated via the honey bee dopamine receptors *Am*DOP1 [26] and *Am*DOP3 [5], and the honey bee tyramine receptor, *Am*TYR1 [27]. Our results are shown in Figure 7 and summarized in Table 1. We found that mianserin acted as a relatively weak antagonist on the *Am*TYR1 receptor and that spiperone, also in relatively high concentrations, blocked cAMP responses mediated via *Am*DOP1. Responses mediated via *Am*DOP3 receptors were not affected significantly by mianserin, epinastine, *cis*-(Z)-flupentixol or spiperone.

**Table 1:** Estimated IC_50_ values of antagonists on cAMP responses mediated via honeybee biogenic amine receptors.

ANTAGONIST	RECEPTOR				
	*Am*DOP1	*Am*DOP2	*Am*DOP3	*Am*OA1	*Am*TYR1
***cis*** **-(Z)-Flupentixol**	NS	0.3 µM	NS	1.3 µM	NS
**Spiperone**	90 µM	10.8 µM	NS	12.5 µM	NS
**Mianserin**	NS	0.4 µM	NS	4.1 µM	73 µM
**Epinastine**	NS	1.1 µM	NS	3.3 µM	NS

Estimated IC_50_ values were calculated by non-linear regression using GraphPad Prism software for Macintosh version 5.0b using data displayed in Figures 5 and 7. NS represents a finding of no significant antagonist activity.

### Selectivity of epinastine

**Figure 8:**
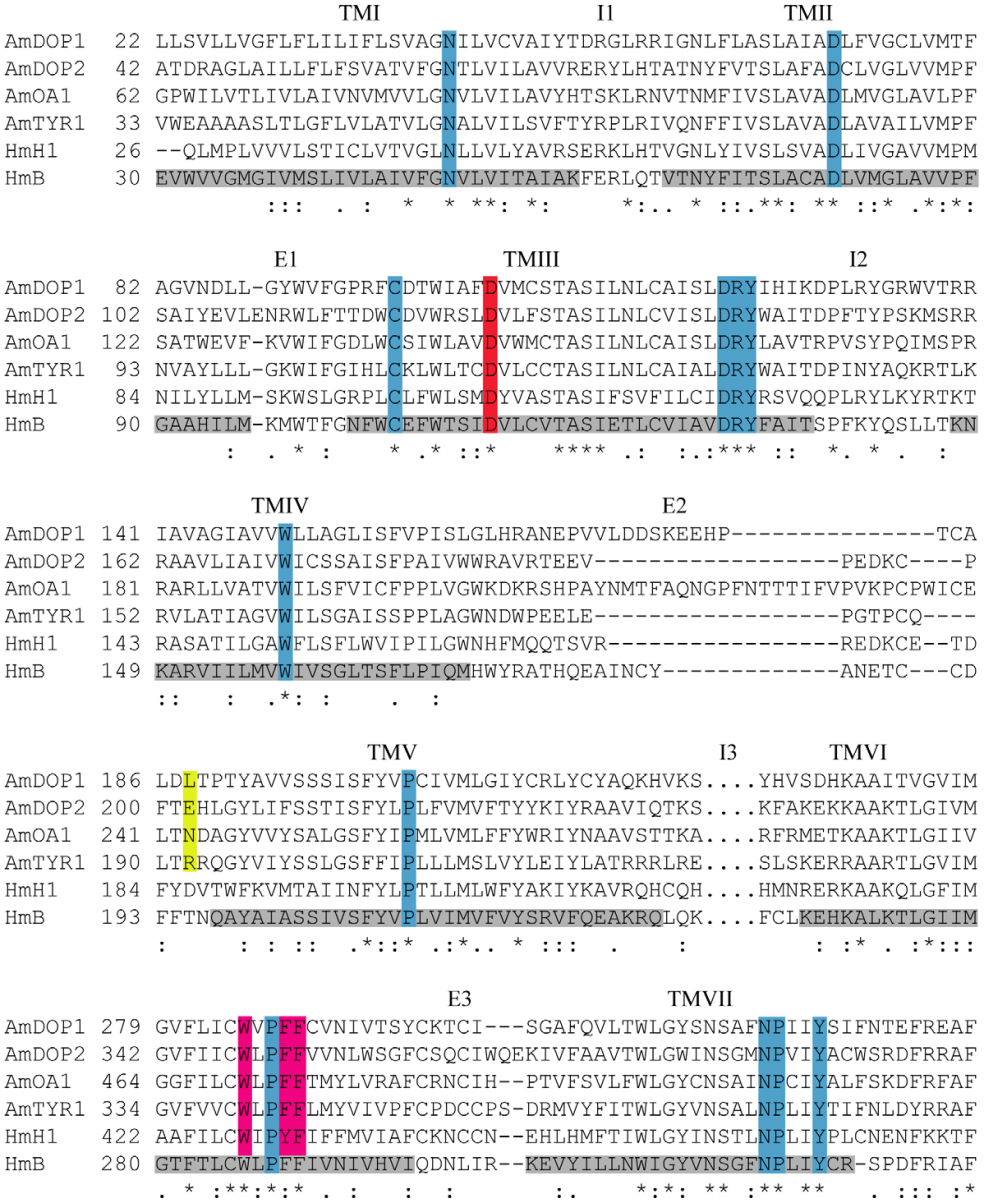
Sequence alignment of *Am*DOP1, *Am*DOP2, *Am*OA1, AmTYR1, human H1 histamine receptor (NP_001091683.1), human β-adrenergic receptor (NP_000015.1). Residues highlighted in gray represent the transmembrane helices from the structure of the human β-adrenergic receptor (pdb2rh1), residues highlighted in cyan are those conserved in the GPCR family [43], the red aspartic acid is the highly conserved D107 (hHis numbering: Asp113 - HmB-Adr) on helix 3 (TMIII; residues not shown for I3).

Analysis of putative ligand binding sites reveals only subtle differences between *Am*DOP1, *Am*DOP2 and *Am*OA1 (Figure 8). Three aromatic residues are completely conserved in TM6 (W285, F288 and F289; *Am*DOP1 numbering). Why then does the antihistamine, epinastine, bind both to *Am*DOP2 and *Am*OA1 but not to *Am*DOP1? A structural analysis of homology models reveals *Am*DOP2, *Am*OA1 and *Am*TYR1 possess a hydrophilic residue (E201, N243 and R192, respectively, yellow Figure 8) prior to TMV whereas *Am*DOP1 has a leucine residue (L188). The corresponding position (D186) has been previously shown to be important for histamine binding to H1 receptors (see Discussion).

## Discussion

Phylogenetic analyses indicate that invertebrate-type DA receptors are more closely related to α-adrenergic-like OA receptors than to vertebrate D1-like receptors [17], [18], see Figure 1. However, while phylogenetic models have proven very useful for the reliable identification of conserved GPCR receptor ortholog families, limitations inherent in such models restrict their utility for confident identification of evolutionary relationships between GPCR receptors [24], [28]. Direct comparison in this study of the functional properties of the invertebrate-type DA receptor, *Am*DOP2, and the α-adrenergic-like OA receptor, *Am*OA1, enabled us to determine whether the functional properties of these two receptor types are indeed conserved.

### *Am*DOP2 and *Am*OA1 receptors exhibit similar functional properties

Previous studies have shown that activation of the honey bee DA receptor, *Am*DOP2, leads to a rise in intracellular levels of cAMP [7], [29], [30], and our results indicate that this invertebrate-type DA receptor couples also to intracellular Ca^2+^ signaling pathways (Figure 2). The *Drosophila* ortholog of the *Am*DOP2 receptor (the DAMB/DopR99B receptor [9], [8]) has been found to show similar properties [9], [14], [15]. For example, expressed in HEK293 cells, DopR99B/DAMB receptors respond to DA not only with an increase in intracellular cAMP, but also with a rapid, transient rise in intracellular Ca^2+^ levels [14], and activation of DAMB/DopR99B receptors expressed in *Xenopus* oocytes results in transient activation of an endogenous Ca^2+^-dependent chloride current [9], [15].

The ability to interact with both cAMP and Ca^2+^ signaling pathways has been reported also for several invertebrate α-adrenergic-like OA receptor orthologues [22], [23], including the honey bee receptor, *Am*OA1 [21]. However, in marked contrast to the invertebrate-type DA receptor, *Am*DOP2, and the adrenergic-like OA receptor, *Am*OA1, we could find no evidence that the honey bee DA receptors, *Am*DOP1 or *Am*DOP3, interact with calcium-signaling pathways, as well as with cAMP. This was true also for the honey bee tyramine receptor, *Am*Tyr1, a result consistent with the findings of Blenau et al. [27]. Thus, in terms of their coupling to second-messenger systems, the functional properties of *Am*DOP2 receptors appear to be more similar to those of *Am*OA1 receptors than to those of *Am*DOP1, *Am*DOP3 or *Am*Tyr1 receptors.

### *Am*DOP2 receptors show independent coupling to two signaling pathways

Previous studies have shown that depleting intracellular Ca^2+^ levels with BAPTA-AM has no significant effect on changes in intracellular cAMP mediated either via the *Drosophila* DA receptor, DAMB/DopR99B [15], or the cockroach α-adrenergic-like OA receptor, Pa oa_1_ [22]. These results suggest that these receptor types mediate their effects on intracellular cAMP, independent of Ca^2+^ signaling [15], [22]. However, this conclusion has been challenged with the alternative explanation that increases in intracellular cAMP levels are a secondary effect of receptor activation, resulting from receptor-mediated increases in intracellular Ca^2+^ inducing adenylyl cyclase activity [16], [21].

Studies of the Dop99B/DAMB receptor [9], [15] have suggested that this invertebrate-type DA receptor is most likely to be coupled to phosphoinositide metabolism via the activity of PLCβ, with receptor activation giving rise to rapid increases in intracellular Ca^2+^ levels. In an attempt to determine whether *Am*DOP2 and *Am*OA1 receptors couple to two independent signaling pathways, we tested the effect of the PLCβ-specific inhibitor edelfosine on responses mediated via *Am*DOP2 and *Am*OA1. Results showing that edelfosine treatment significantly reduces *Am*DOP2- and *Am*OA1-receptor-mediated Ca^2+^ signaling (Figure 3), indicate the involvement of PLCβ activity in the Ca^2+^ signals initiated by the activation of these receptors. Interestingly, *Am*DOP2- and *Am*OA1-receptor-mediated increases in intracellular levels of cAMP were not affected by edelfosine (Figure 4). While this result does not rule out the possibility that adenylyl cyclase activity is affected by changes in intracellular Ca^2+^ levels, it suggests that *Am*DOP2- and *Am*OA1-receptor-mediated cAMP signaling is not dependent on receptor-mediated changes in intracellular Ca^2+^.

It will be of considerable interest in the future to clarify the mechanism of G-protein coupling of *Am*DOP2 and *Am*OA1 receptors to cAMP- and Ca^2+^-mediated signaling, and the relative importance of these two signaling pathways *in vivo*. The coupling of the DAMB/Dop99B receptor to specific classes of heterotrimeric G proteins has been investigated in *Xenopus* oocytes [15]. Unusually, it was found that cAMP signalling via this receptor was inhibited by pertussis toxin, suggesting coupling to the G_i/o_ class of G-proteins and signaling by G_βγ_ subunits. In contrast DAMB/Dop99B-mediated Ca^2+^ signaling was found not to be sensitive to pertussis toxin treatment nor to involve signaling via G_βγ_ subunits. These results were suggested to indicate that this receptor is able to couple to multiple G-protein effectors, potentially mediating its effects on Ca^2+^ by coupling to the G_q_ class of subunits [15]. Interestingly, a study of the orthologous D_1αPan_ receptor in the spiny lobster found evidence for coupling to the G_s_ class of G-proteins, but neither the G_q_ or G_i/o_ classes [11]. To date there have been no reports on the G-protein coupling of any member of the α-adrenergic-like OA receptors.

### *Am*DOP2 and *Am*OA1 share similar pharmacological properties

GPCR receptors with a close evolutionary relationship are frequently found to display similarities in their pharmacological properties [24] and in the present study we found this to be true of *Am*DOP2 and *Am*OA1. Interestingly, c*is*-(Z)-flupentixol and spiperone, compounds generally used as dopamine receptor antagonists in insects [1], [29], as well as mianserin and epinastine, which are known to function as invertebrate OA receptor antagonists [20], [23], [31], were all found to be effective blockers of *Am*DOP2 receptors, as well as *Am*OA1 receptors (Figure 5). Comparison of the estimated IC_50_ values (Table 1) indicates that the rank order of potency of these antagonists on the *Am*DOP2 receptor is as follows: *cis*-(Z)-flupentixol≥mianserin>epinastine>spiperone, and on the *Am*OA1 receptor, *cis*-(Z)-flupentixol>epinastine≥mianserin>spiperone. All four compounds were also found to be highly effective at blocking *Am*DOP2- and *Am*OA1-mediated Ca^2+^ signaling (Figure 6). Indeed, our data suggest that spiperone, and to a lesser extent cis-(Z)-flupentixol, may be more potent blockers of Ca^2+^ signals mediated via *Am*OA1 and *Am*DOP2 than cAMP responses (compare Figures 5 and 6), however further analysis is required to confirm this finding. In contrast, we found that the same compounds were significantly less effective at blocking activation of the DA receptors, *Am*DOP1 and *Am*DOP3, and the tyramine receptor, *Am*TYR1 (Table 1 & Figure 7). Consistent with earlier studies [29], we found that spiperone exhibited significant antagonist activity on the D1-like DA receptor, *Am*DOP1 and we found in addition that mianserin blocked activity mediated via the *Am*TYR1 receptor. However, both antagonists were more effective on *Am*DOP2 and *Am*OA1 receptors than on *Am*DOP1, *Am*DOP3 or *Am*TYR1.

### *Cis*-(Z)-flupentixol

We were surprised by our finding that *cis*-(Z)-flupentixol had no significant antagonist activity on either, *Am*DOP1 or *Am*DOP3 receptors (Figure 7, Table 1). This was unexpected because *cis*-(Z)-flupentixol has been found in earlier studies to be an effective *Am*DOP1 receptor antagonist (26,29) and is reported in the fruit fly to block *Dm*Dop1/dDA1 [32], [33], DAMB/Dop99B [15] and DD2R [4]. It is reported also to be an effective blocker of the two D1-like DA receptors found in the silkworm [13], and a highly effective reverse agonist, not only of the honey bee *Am*DOP1 receptor [29], but also of the orthologous receptors in *Aplysia* (*Ap*Dop1) and *C. elegans* (dop-1) [34], [35]. It is possible that our use of an indirect cAMP reporter system in this study contributed to our inability to detect antagonist activity of *cis*-(Z)-flupentixol on *Am*DOP1. Our data indicate, however, that *cis*-(Z)-flupentixol is less effective at blocking *Am*DOP1 receptors than *Am*DOP2 receptors, a result that is consistent with earlier studies [1], [29]. In combination these results suggest the *in vivo* effects of *cis*-(Z)-flupentixol treatment in insects are complex, not confined to dopaminergic signaling and potentially, species specific.

### Spiperone

Consistent with earlier studies on honey bee dopamine receptors [26], [29] we found spiperone to be an effective antagonist at both of the honey bee D1-like dopamine receptors, *Am*DOP1 (Figure 7) and *Am*DOP2 (Figure 4B). Interestingly, spiperone also acts as an antagonist at the honey bee octopamine receptor, *Am*OA1 (Figure 5B), but is not an effective blocker of the D2-like dopamine receptor, *Am*DOP3 (Figure 7). This is consistent with studies of DA receptors in *Drosophila*, where spiperone has been reported to be an antagonist of *Drosophila* D1-like DA receptors, *Dm*Dop1 [33] and DAMB [8], but not the *Drosophila* D2-like receptor, DD2R [4]. These results are quite different to those reported for vertebrate DA receptors as in vertebrates, spiperone acts as a selective D2-like DA receptor blocker [3].

### Mianserin and epinastine

Mianserin has previously been found to be an antagonist of three *Drosophila* ‘β-adrenergic-like’ OA receptors [36], and the silkworm α-adrenergic-like OA receptor, *Bm*OAR [23]. Mianserin has also been shown to have a significant affinity for the *Drosophila* tyramine receptor, *Dm*TyrR [37], and in *C. elegans*, this antagonist is reported to block the serotonin receptors, SER-3 and SER-4 [38]. In this study we found that mianserin blocked responses mediated via *Am*OA1, *Am*DOP2 and *Am*TYR1 receptors, and that epinastine was an effective antagonist not only of the honey bee OA receptor, AmOA1, but also the DA receptor, *Am*DOP2. Taken together, these results suggest that *in vivo* effects of treatment with either mianserin or epinastine are unlikely to be confined to octopaminergic targets, as has previously been suggested [31], [39]. Nonetheless, epinastine may prove useful in future studies for differentiating between responses mediated via *Am*DOP1 and *Am*DOP2, as *Am*DOP2 receptors are blocked very effectively by this antagonist, whereas *Am*DOP1 receptors are not. *Am*DOP2 and *Am*OA1 share a hydrophilic residue prior to TMV (E201 and N243 respectively, yellow alignment, Figure 8). In the corresponding position (L188) *Am*DOP1 has a leucine residue. This difference may help to explain the activity of epinastine at *Am*DOP2 and *Am*OA1 but not *Am*DOP1 as in human H1 receptors, the corresponding position (D186) has been shown to be important for histamine binding [40]. Interestingly however, responses mediated via AmTYR1, which possesses a hydrophilic but positively charged amino acid at the position (R192), were not affected by epinastine.

### *Amdop2* and *Amoa1*: immediate paralogs?

Our results show that of the five receptors examined, *Am*DOP2 and *Am*OA1 are the most similar in terms of their functional properties and pharmacological profile. This evidence suggests to us that the evolutionary relationship between these two receptor types is most likely to be that of immediate paralogs, and that despite divergence in their native ligand specificities, invertebrate-type DA receptors and α-adrenergic-like OA receptors still display significant conservation in their functional properties. The results of this study highlight the need to identify antagonists that act selectively on specific invertebrate receptor types. The identification of such compounds would greatly assist studies exploring the *in vivo* function(s) of biogenic amine receptors in invertebrates.

## Materials and Methods

### Materials

Dopamine hydrochloride, DL-octopamine hydrochloride, *cis*-(Z)-flupenthixol dihydrochloride, spiperone, epinastine hydrochloride and mianserin hydrochloride were obtained from Sigma-Aldrich. Edelfosine (2-O-methyl-PAF C-18) was obtained from Cayman Chemical Company, Ann Arbor, U.S.A.

### Heterologous expression of the honey bee receptor proteins

HEK293 cells (Invitrogen) were maintained as adherent cultures at 37°C, 5% CO_2_ in phenol-red free DMEM/F12 medium containing 10% fetal calf serum (Invitrogen). For expression of the receptor proteins in HEK293 cells, expression plasmid constructs were transiently transfected into the cells using FuGene-HD reagent (Roche) in accordance with the manufacturer's instructions. Control cells transfected with pIB/V5-His-GW/LacZ for expression of the beta-galactosidase reporter protein indicated that transfection efficiency was >95%.

The creation of plasmid expression constructs for *Am*DOP1, *Am*DOP2, *Am*DOP3 and *Am*OA1 has been described in detail elsewhere [29]. The expression construct for the *Am*TYR1 receptor was created by PCR amplification of the coding sequences of the *Amtyr1* cDNA [27] and insertion into the HindIII and XbaI site of pcDNA3.1(+) vector using the following primer sequences; forward – GCACGAAGCTTGCCACCATGAACTCGAGCGGGGAATCAG; reverse – GACTTCTAGATCAACGAATGCGCAACAACCGTCT.

### Measurement of [Ca^2+^]_i_ levels for assaying receptor function

Exponentially growing HEK293 cells (Invitrogen) were dispensed at a density of 2×10^4^ cells per well in 96-well, black-walled, clear-bottomed tissue culture plates (Greiner Bio-One) and allowed to grow for 24 hours. The cells were transfected with the desired honey bee receptor expression construct. Transfected cells were maintained for a further 24 hours at 37°C prior to assaying for receptor function. Intracellular Ca^2+^ levels were assayed by preloading the cells with Fluo-4 NW reporter dye dissolved in Hank's buffer in accordance with the manufacturer's instructions (Invitrogen). The fluorescence signal (excitation 480 nm, emission 520 nM) of individual wells was detected using a BMG Labtech Fluostar Omega microplate instrument. Amines were prepared immediately prior to use in Hanks buffer, and a 2 ul volume was introduced into test wells using onboard injectors. Treatment concentrations indicated in figure legends represent the final concentration of amine in the test well. Wells were monitored for fluorescent signal immediately prior to treatment to establish the baseline fluorescence (F_b_) and for 50 seconds post agonist injection to record changes in fluorescence (ΔF) at 0.24 second intervals. Amines added to test wells remained in the medium throughout the post-injection recording period.

### Indirect measurement of intracellular cAMP levels for assaying receptor function

Receptor coupling to intracellular cAMP signaling was assessed using a CRE-luciferase reporter as detailed previously [29]. In brief, HEK293 cells were co-transfected with the desired honey bee receptor expression construct together with the pGL4.29[luc2P/CRE/Hygro] reporter construct (Promega) and grown for a further 24 hours. The amount of *Am*DOP1 expression construct used for transfections was reduced to 1/10^th^ of the concentration used in earlier studies [29], because high-level expression of this constitutively active receptor [28] was found to swamp the capacity of the reporter system [41]. Cells were then incubated for 3 hours in serum-free growth medium containing the test treatments detailed in figure legends, and then immediately assayed for luciferase enzyme activity. Duplicate measurements were determined for each test treatment examined in the performance of independent trials. All *in vitro* expression work was conducted under approvals issued by the University of Otago Institutional Biological Safety Committee.

### Sequence alignment and homology modelling

The sequence alignment was initially carried out using T-coffee and then manually adjusted in a similar fashion to that described elsewhere [42]. Models were generated using Modeller9v7 [43] using the human beta-adrenergic structure (pdb2rh1) as a template. The models with the lowest objective function were selected for further analysis. Docking experiments were carried out using Gold 4.1 [44] to dock the epinastine into the binding site of the human histamine receptor. Both isomers of epinastine were used in the docking calculations and were downloaded from the Cambridge Crystallographic Data Centre (ID: CALQUC: *R*, CALRAJ; *S*).

## References

[1] Mustard JA, Beggs KT, Mercer AR (2005). Molecular biology of the invertebrate dopamine receptors.. Arch Insect Biochem Physiol.

[2] Mcdonald P, Jessen T, Field J, Blakely R (2006). Dopamine signaling architecture in *Caenorhabditis elegans*.. Cell Mol Neurobiol.

[3] Missale C, Nash SR, Robinson SW, Jaber M, Caron MG (1998). Dopamine receptors: from structure to function.. Physiol Rev.

[4] Hearn MG, Ren Y, McBride EW, Reveillaud I, Beinborn M (2002). A *Drosophila* dopamine 2-like receptor: Molecular characterization and identification of multiple alternatively spliced variants.. Proc Natl Acad Sci USA.

[5] Beggs KT, Hamilton IS, Kurshan PT, Mustard JA, Mercer AR (2005). Characterization of a D2-like dopamine receptor (*Am*DOP3) in honey bee, *Apis mellifera*.. Insect Biochem Mol Biol.

[6] Clark MC, Baro DJ (2007). Arthropod D2 receptors positively couple with cAMP through the Gi/o protein family.. Comp Biochem Physiol B Biochem Mol Biol.

[7] Humphries MA, Mustard JA, Hunter SJ, Mercer A, Ward V (2003). Invertebrate D2 type dopamine receptor exhibits age-based plasticity of expression in the mushroom bodies of the honeybee brain.. J Neurobiol.

[8] Han KA, Millar NS, Grotewiel MS, Davis RL (1996). DAMB, a novel dopamine receptor expressed specifically in *Drosophila* mushroom bodies.. Neuron.

[9] Feng G, Hannan F, Reale V, Hon YY, Kousky CT (1996). Cloning and functional characterization of a novel dopamine receptor from *Drosophila melanogaster*.. J Neurosci.

[10] Ono H, Yoshikawa H (2004). Identification of amine receptors from a swallowtail butterfly, *Papilio xuthus* L.: cloning and mRNA localization in foreleg chemosensory organ for recognition of host plants.. Insect Biochem Mol Biol.

[11] Clark MC, Baro DJ (2006). Molecular cloning and characterization of crustacean type-one dopamine receptors: D1alphaPan and D1betaPan.. Comp Biochem Physiol B, Biochem Mol Biol.

[12] Gerber S, Krasky A, Rohwer A, Lindauer S, Closs E (2006). Identification and characterisation of the dopamine receptor II from the cat flea *Ctenocephalides felis* (CfDopRII).. Insect Biochem Mol Biol.

[13] Ohta H, Tsuchihara K, Mitsumasu K, Yaginuma T (2009). Comparative pharmacology of two D1-like dopamine receptors cloned from the silkworm *Bombyx mori*.. Insect Biochem Mol Biol.

[14] Han KA, Millar NS, Davis RL (1998). A novel octopamine receptor with preferential expression in *Drosophila* mushroom bodies.. J Neurosci.

[15] Reale V, Hannan F, Hall LM, Evans PD (1997). Agonist-specific coupling of a cloned *Drosophila melanogaster* D1-like dopamine receptor to multiple second messenger pathways by synthetic agonists.. J Neurosci.

[16] Balfanz S, Strunker T, Frings S, Baumann A (2005). A family of octopamine receptors that specifically induce cyclic AMP production or Ca2+ release in *Drosophila melanogaster*.. J Neurochem.

[17] Hauser F, Cazzamali G, Williamson M, Blenau W, Grimmelikhuijzen CJ (2006). A review of neurohormone GPCRs present in the fruitfly *Drosophila melanogaster* and the honey bee *Apis mellifera*.. Prog Neurobiol.

[18] Hauser F, Cazzamali G, Williamson M, Park Y, Li B (2007). A genome-wide inventory of neurohormone GPCRs in the red flour beetle *Tribolium castaneum*.. Front Neuroendocrinol.

[19] Evans PD, Maqueira B (2005). Insect octopamine receptors: a new classification scheme based on studies of cloned *Drosophila* G-protein coupled receptors.. Invert Neurosci.

[20] Gerhardt CC, Bakker RA, Piek GJ, Planta RJ, Vreugdenhil E (1997). Molecular cloning and pharmacological characterization of a molluscan octopamine receptor.. Mol Pharmacol.

[21] Grohmann L, Blenau W, Erber J, Ebert PR, Strunker T (2003). Molecular and functional characterization of an octopamine receptor from honeybee (*Apis mellifera*) brain.. J Neurochem.

[22] Bischof LJ, Enan EE (2004). Cloning, expression and functional analysis of an octopamine receptor from *Periplaneta americana*.. Insect Biochem Mol Biol.

[23] Ohtani A, Arai Y, Ozoe F, Ohta H, Narusuye K (2006). Molecular cloning and heterologous expression of an alpha-adrenergic-like octopamine receptor from the silkworm *Bombyx mori*.. Insect Mol Biol.

[24] Vernier P, Cardinaud B, Valdenaire O, Philippe H, Vincent JD (1995). An evolutionary view of drug-receptor interaction: the bioamine receptor family.. Trends Pharmacol Sci.

[25] Powis G, Seewald MJ, Gratas C, Melder D, Riebow J (1992). Selective inhibition of phosphatidylinositol phospholipase C by cytotoxic ether lipid analogues.. Cancer Research.

[26] Blenau W, Erber J, Baumann A (1998). Characterization of a dopamine D1 receptor from *Apis mellifera*: cloning, functional expression, pharmacology, and mRNA localization in the brain.. J Neurochem.

[27] Blenau W, Balfanz S, Baumann A (2000). Amtyr1: characterization of a gene from honeybee (*Apis mellifera*) brain encoding a functional tyramine receptor.. J Neurochem.

[28] Baldauf S (2003). Phylogeny for the faint of heart: a tutorial.. Trends Genet.

[29] Mustard JA, Blenau W, Hamilton IS, Ward VK, Ebert PR (2003). Analysis of two D1-like dopamine receptors from the honey bee *Apis mellifera* reveals agonist-independent activity.. Mol Brain Res.

[30] Beggs KT, Mercer AR (2009). Dopamine receptor activation by honey bee queen pheromone.. Curr Biol.

[31] Roeder T, Degen J, Gewecke M (1998). Epinastine, a highly specific antagonist of insect neuronal octopamine receptors.. Eur J Pharmacol.

[32] Gotzes F, Balfanz S, Baumann A (1994). Primary structure and functional characterization of a *Drosophila* dopamine receptor with high homology to human D1/D5 receptors.. Receptors Channels.

[33] Sugamori KS, Demchyshyn LL, McConkey F, Forte MA, Niznik HB (1995). A primordial dopamine D1-like adenylyl cyclase-linked receptor from *Drosophila melanogaster* displaying poor affinity for benzazepines.. FEBS Lett.

[34] Barbas D, Zappulla JP, Angers S, Bouvier M (2006). An *Aplysia* dopamine-like receptor: molecular and functional characterization.. J Neurochem.

[35] Sanyal S, Wintle RF, Kindt KS, Nuttley WM (2004). Dopamine modulates the plasticity of mechanosensory responses in *Caenorhabditis elegans*.. EMBO J.

[36] Maqueira B, Chatwin H, Evans PD (2005). Identification and characterization of a novel family of *Drosophila* β-adrenergic-like octopamine G-protein coupled receptors.. J Neurochem.

[37] Enan EE (2005). Molecular response of *Drosophila melanogaster* tyramine receptor cascade to plant essential oils.. Insect Biochem Mol Biol.

[38] Petrascheck M, Ye X, Buck LB (2007). An antidepressant that extends lifespan in adult *Caenorhabditis elegans*.. Nature.

[39] Degen J, Gewecke M, Roeder T (2000). Octopamine receptors in the honey bee and locust nervous system: pharmacological similarities between homologous receptors of distantly related species.. Br J Pharmacol.

[40] Gantz I, DelValle J, Wang L, Tashiro T, Munzert G (1992). Molecular basis for the interaction of histamine with the histamine H2 receptor.. J Biol Chem.

[41] Hill SJ, Baker JG, Rees S (2001). Reporter-gene systems for the study of G-protein-coupled receptors.. Curr Opinion Pharmacol.

[42] Bissantz C, Bernard P, Hibert M, Rognan D (2003). Protein-based virtual screening of chemical databases. II. Are homology models of G-Protein Coupled Receptors suitable targets?. Proteins.

[43] Fiser A, Šali A, Carter CWJ PartD, Sweet RM (2003.). Methods in Enzymology: Macromolecular Crystallography.

[44] Jones G, Willett P, Glen RC, Leach AR, Taylor RJ (1997). Development and validation of a genetic algorithm for flexible docking.. Mol Biol.

